# Pleiotropic effects on cardiovascular risk factors within and between the fourth and sixth decades of life: Implications for genotype × age interactions

**DOI:** 10.1186/1471-2156-4-S1-S54

**Published:** 2003-12-31

**Authors:** LM Havill, MC Mahaney

**Affiliations:** 1 Department of Genetics, Southwest Foundation for Biomedical Research, San Antonio, Texas, 78227, USA

## Abstract

We used an approach for detecting genotype × environment interactions to detect and characterize genotype × age interaction in longitudinal measures of three well known cardiovascular risk factors: total plasma cholesterol (TC), systolic blood pressure (SBP), and body weight (Wgt). Our objectives were to determine if the same gene or suite of genes influences quantitative variation in each of these phenotypes in the 4^th ^and 6^th ^decades of life, to assess the impact of additive gene effects in these two decades, and to evaluate the stability of pleiotropic relationships among these phenotypes. Using the Framingham Heart Study data, we constructed two cross-sectional samples comprising individuals on whom these phenotypes were measured at ages 30-39 years (Original Cohort: exam 1, Offspring Cohort: exam 2) and at ages 50-59 years (Original Cohort: exam 11, Offspring Cohort: exam 5). We also constructed a longitudinal sample from the cross-sectional sample members for whom measures on these traits were available at both ages (i.e., 4^th ^and 6^th ^decades of life). Patterns of pleiotropy, inferred from genetic correlations between traits, differ between the two age classes. Further, additive genetic variance in SBP during the 4^th ^decade of life is attributable to a different gene or suite of genes than during the 6^th^. The magnitude of the effect increases for SBP. Variation in TC and Wgt appear to be influenced by the same gene or genes in both decades. The magnitude of the effect is stable for TC, but increases dramatically with age for Wgt.

## Background

Longitudinal data, limited in the past primarily to human twin studies, are currently being developed by some large-scale family studies entering their second or greater multiyear study cycles, as well as from long-term epidemiological studies that have recruited relatives to facilitate pedigree construction and statistical genetic analyses. Using data from one such long-term epidemiological study, the Framingham Heart Study, we present the results of analyses using an approach for detecting genotype × environment interactions to detect and characterize genotype × age interaction in longitudinal measures of three well known cardiovascular risk factors: total plasma cholesterol, systolic blood pressure, and body weight.

The rationale for the approach used here has been described in detail elsewhere [1,2]. Briefly, we modeled the different ages as two discrete environments in which all individuals are measured. Using multivariate variance decomposition methods we estimated both the genetic variances for the traits in the two age "environments" and the genetic correlations between the same trait measured in the two age "environments." Assuming no genotype-by-age interaction, the genetic variances for the same trait at the two ages should be equal and the genetic correlations between the trait at the two ages should equal 1.0 or -1.0. Departures from these conditions (i.e., unequal genetic variances and incomplete pleiotropy) are interpreted to indicate genotype × age interaction.

The primary objectives of our analyses for Genetic Analysis Workshop 13 (GAW13) were 1) to determine if the same gene or suite of genes influences quantitative variation in these three cardiovascular risk factors in the 4^th ^and 6^th ^decades of life, 2) to determine if the impact of these genes, as measured by the additive genetic variance for these three cardiovascular risk factors, changes in these two decades, and 3) to determine if pleiotropic relationships between these variables differ between these two decades of life.

## Methods

The Framingham data set for GAW13 includes data on various cardiovascular risk factors for two cohorts, the Original Cohort, recruited in 1948, and the Offspring Cohort, recruited in 1971. Members of the Original Cohort were measured every 2 years. Members of the Offspring Cohort were measured every 4 years (except that the first and the second exams were 8 years apart). For this analysis we used exams for both cohorts that were 20 years apart.

### Sample and sub-samples

#### Cross-sectional sample

To construct this sample, we selected the subset of all individuals in the Original Cohort who were 30-39 years of age or 50-59 years of age at exams 1 and 11, and all Offspring Cohort individuals who were 30-39 years of age or 50-59 years of age at exams 2 and 5. These individuals from the two cohorts were organized into two Framingham Heart Study pedigree sub-samples: one containing families comprising individuals for whom all measurements were obtained in the 4^th ^decade of life and the other containing families comprising individuals whose measurements were obtained in the 6^th ^decade of life.

#### Longitudinal Sample

This sample consists of the subset of individuals from the cross-sectional sample described above for whom data were available at both ages (in their 30s, and again, 20 years later, in their 50s).

### Data: phenotypes and covariates

The three traits, total cholesterol (TC), systolic blood pressure (SBP), and body weight (Wgt), meet the following criteria: data on them were available in both the Original and Offspring cohorts and available in a sufficient number of individuals in two specific age classes, 20 years apart (30-39 years and 50-59 years), to support a statistical genetic analysis with reasonable power. Similarly, data were available for five potential covariates (sex, age, cigarettes per day (CPD), hypertension medications (HRx), and (in some cases) weight (Wgt)) in a majority of individuals, ages 30-39 years and 50-59 years, from exams 1 and 11 for the Original Cohort and from exams 2 and 5 from the Offspring Cohort.

### Statistical genetic analyses

All pedigree data management was accomplished using routines in the computerized pedigree data management software PEDSYS (B. Dyke, Southwest Foundation for Biomedical Research). All statistical genetic analyses were conducted using maximum likelihood-based variance decomposition approaches implemented in SOLAR [3].

#### Analyses of data from cross-sectional pedigree samples

For each of the two age-class pedigree data sets, basic univariate quantitative genetic analyses were used to simultaneously estimate the proportions of the phenotypic variance in each of the three phenotypes attributable to the additive effects of genes and the random environment, and the mean effects on these phenotypes of the potential covariates. The phenotypic covariance within the pedigrees was modeled as: , where  is the additive genetic variance multiplied by twice the kinship matrix and  is the random environmental component to the variance multiplied by the identity matrix. Each phenotype was modeled as , i.e., the sum of the phenotypic mean (μ), plus the mean effects of *n *significant covariates (), genes (*g*), and random environmental factors (*e*). Significance was determined by likelihood ratio tests. Descriptions of these tests, including determination of appropriate degrees of freedom, are provided elsewhere [3].

We tested for genotype × age interactions by hypothesizing that an individual's age constitutes an environmental milieu in which a phenotype is expressed [4]. Using multivariate extensions to the variance decomposition procedures implemented in SOLAR [3], we reparameterized our basic quantitative genetic model to simultaneously estimate means, covariate effects, residual additive genetic and environmental standard deviations for each of the two traits in a trait pair, plus the additive genetic and random environmental correlations between the two traits. The former, ρ_G_, is an estimate of the proportion of additive genetic variance shared between trait pairs and the latter, ρ_E_, is an estimate of the proportion of the residual environmental variance due to shared effects of unmeasured environmental factors within each age class. Like their analogous components of the phenotypic covariance, these components of the phenotypic correlation (ρ_P_) are additive, so that . Significance of these parameters was determined by likelihood ratio tests. Genetic correlations differing from zero indicate pleiotropy (shared additive genetic effects on a trait pair). Significant genetic correlations differing from 1.0 or -1.0 indicate incomplete pleiotropy (i.e., less than 100% of the genetic variance in both traits is due to the effects of the same gene(s)). The squared genetic correlation is an estimate of the proportion of the additive genetic variance in each trait of the pair that is due to the effects of the same gene(s).

#### Analyses of data from longitudinal pedigree data sample

We also conducted bivariate quantitative genetic analyses with longitudinal data from a subset of individuals for whom measures on all three phenotypes and potential covariates were available from exams 20 years apart. We used this approach to estimate genetic and environmental correlations between phenotypes measured in the same individuals in the 4^th ^and 6^th ^decades of life. We used likelihood ratio tests to detect departures from complete pleiotropy.

We interpret different genetic variances for the same trait between decades as further evidence of genotype × age interaction, implying different levels of gene expression at different ages. We obtained the genetic variance as a product of the proportion of the total phenotypic variance due to the additive effects of genes and the total phenotypic variance for each trait at each age.

## Results

Results from univariate quantitative genetic analyses of the three phenotypes in the cross-sectional samples from the two age classes are summarized in Table 1. We estimated the effects of sex, age, CPD, HRx, and Wgt (where appropriate) to increase the genetic signal-to-noise ratio for each trait, rather than testing specific hypotheses about the effects of those variables. In general, the proportion of the phenotypic variance attributable to these covariates (i.e., c^2 ^in this paper) decreases with age and, with one exception (SBP), sex contributes significantly to variation in each trait in both decades of life. Because each age class spans 10 years, the effect of within-decade age variation on each phenotype was also estimated.

The estimated heritabilities for both TC and Wgt decrease with age (not significant, *p *= 0.46 and *p *= 0.28, respectively) and that for SBP increases (*p *= 0.002). When these residual values were re-scaled as a proportion of the total phenotypic variance, the heritabilities in the earlier and later age-class samples are, respectively, for TC, 0.32 and 0.28 (12.5% decrease); for SBP, 0.09 and 0.31 (greater than two-fold increase); and for Wgt, 0.29 and 0.33 (13% increase).

A summary of the bivariate quantitative genetic analyses of trait pairs *within *the two age classes is presented in Table 2. Residual heritability estimates for the two age classes are similar to those obtained in the univariate analyses of these data. The patterns of pleiotropy, inferred from the genetic correlations, appear to differ between the two age classes. The additive genetic correlation between TC and SBP increases more than five-fold in the older age class and that between SBP and Wgt decrease to a similar degree in the older individuals. While the magnitude of the genetic correlation between TC and Wgt does not change in the older sample, the direction does change from negative to positive. Overall, these genetic correlations are small to moderate, suggesting that shared genetic effects account for 1% to 19% of the additive genetic variance in the trait pairs in 30- to 39-year-olds (mean = 9%) and 1% to 24% in the 50- to 59-year-olds (mean = 11%).

Bivariate quantitative genetic analyses of the longitudinal data (same individuals, two age points) are summarized in Table 3. Residual heritability estimates for age-specific trait measures are of greater mean magnitude than those estimated from the cross-sectional data, but they exhibit a similar pattern (i.e., modest, nonsignificant decreases in h^2 ^for TC and Wgt, and a larger increase in h^2 ^for SBP as individuals in the sample move from the 4^th ^to the 6^th ^decade of life). When re-scaled to the total phenotypic variance for each age class, the heritability for TC decreases by 21% from the earlier to later decade in our comparison. That for SBP increases by 41%, and that for Wgt increases by 10%. The genetic variances for these three traits show similar changes between the two age classes: i.e., that for TC decreases by 9% (470.9 to 429.2), that for SBP increases by 155% (81.7 to 208.6), and that for Wgt increases by 33% (204.4 to 271.7). The genetic correlation (ρ_G_) between SBP measures in the two decades is significantly less than 1.0 (*p *= 0.009); but those for TC and Wgt are not (*p *= 0.16 and *p *= 0.42, respectively). These results indicate that shared genes account for only 20% of the additive genetic variance in SBP (ρ_G_^2^) at the two ages, but at least 57% of that in TC and at least 96% of that in Wgt.

## Discussion and Conclusion

Studies in humans and nonhuman animals alike strongly support the notion that genes influence normal quantitative variation in these three phenotypes and several studies have shown evidence of pleiotropic interactions (i.e., shared gene effects) between them. Our quantitative genetic analyses of longitudinal data from the Framingham Heart Study pedigrees are consistent with previous observations of others and yield new insights into the likely effects of genotype × age interactions on quantitative variation in these three cardiovascular disease risk factors.

The rejection of the null hypothesis for no genotype × age interaction (|ρ_G_| ≠ 1.0) is unequivocal for SBP, as is the failure to do so for Wgt. However we are less certain about the failure to reject the null hypothesis for TC levels. The standard errors, indicators of relative imprecision around these parameter estimates given this pedigreed data set, are relatively large. The maximum likelihood point estimate of ρ_G _= 0.76 ± 0.24 for TC at the two ages may reflect genuine departures from complete pleiotropy that are undetectable in our analyses of data from this pedigreed sample. This consideration leads us to offer the qualified conclusion that, in the Framingham pedigrees, 57% to 100% of the variance in TC levels in the 4^th ^and 6^th ^decades is due to the effects of the same gene or suite of genes.

The second null hypothesis for no genotype × age interaction is rejected when the genetic variances at two ages are not equal. This is the case for Wgt and for SBP, but the genetic variance is relatively stable for TC. Further, although they do not constitute a formal test for the phenomenon, disruptions of pleiotropic relationships between the three different phenotypes within the two age classes are additional evidence for genotype × age interaction.

These traits provide three potential examples of genotype × age interaction. For TC, the additive genetic variance during the 6^th ^decade of life may be attributable to the same gene or suite of genes as during the 4^th ^decade, and the relative impact of those genes is fairly stable between the two decades. A different gene or suite of genes is responsible for variation in SBP at these two ages and the relative importance of these genes for variation in SBP increases. Lastly, normal quantitative variation in Wgt in this population appears to be influenced by the same gene or genes in both decades of life, but the expression of these genes, inferred from the genetic variances, is increased dramatically in the latter of two decades from which data in this study were obtained.

## Figures and Tables

**Table 1:** Cross-sectional analyses: heritabilities and covariate effects for three traits within age-classes from univariate quantitative genetic analyses of data from Framingham Heart Study subjects measured in the 4^th ^and 6^th ^decades of life.

				*p*-Values of Covariates Screened				
				
Trait	h^2^	c^2A^	n	Sex	Age	CPD	HRx	Wgt
30-39 Years								
Total Cholesterol	0.34	0.06	984	<0.001		<0.001		-^B^
Systolic Blood Pressure	0.10	0.15	994	<0.01		<0.05		<0.001
Body Weight	0.48	0.49	1239	<0.001	<0.01		<0.001	-
50-59 Years								
Total Cholesterol	0.29	0.02	818	<0.001			<0.10	-
Systolic Blood Pressure	0.32	0.04	968		<0.001		<0.05	<0.001
Body Weight	0.45	0.26	881	<0.001		<0.001	<0.001	-

^A^c^2^, proportion of phenotypic variance due to covariates. ^B^-, not screened.

**Table 2:** Cross-sectional analyses: heritabilities and correlations between different traits within age-classes from bivariate quantitative genetic analyses of data from Framingham Heart Study subjects measured in the 4^th ^and 6^th ^decades of life.

	Heritabilities		Correlations^A^			
		
Trait Pair	Trait 1	Trait 2	ρ_G_	ρ_E_	ρ_P_	n
30-39 Years						
TC, SBP	0.34	0.10	-0.09	0.21	0.15	995
TC, Wgt	0.34	0.55	0.27	-0.12	0.05	995
SBP, Wgt	0.10	0.55	0.44	0.23	0.20	995
50-59 Years						
TC, SBP	0.27	0.31	0.49	0.02	0.15	881
TC, Wgt	0.28	0.45	-0.28	0.05	-0.07	882
SBP, Wgt	0.29	0.44	0.11	0.18	0.15	882

^A^ρ_G_, genetic correlation; ρ_E_, environmental correlation; ρ_P_, phenotypic correlation.

**Table 3:** Longitudinal analyses: heritabilities (h^2^), correlations (ρ_G_, ρ_E_, ρ_P_), and proportion of additive genetic variance due to the effects of shared genes (ρ_G_^2^) between the same traits between age-classes from bivariate quantitative genetic analyses of data from subjects measured in both the 4^th ^and 6^th ^decades of life.

	Heritabilities		Correlations^A^				
	
Trait	h^2^_[30–39]_	h^2^_[50–59]_	ρ_G_	ρ_E_	ρ_P_	ρ_G_^2^	n
Total Cholesterol	0.36	0.29	0.76	0.43	0.55	0.58	393
Systolic Blood Pressure	0.50	0.64	0.45	0.23	0.36	0.20	392
Body Weight	0.40	0.30	0.98	0.66	0.77	0.96	393

^A^ρ_G_, genetic correlation; ρ_E_, environmental correlation; ρ_P_, phenotypic correlation.
